# Asthma underdiagnosis in children: A school-based screening in a low socioeconomic status population

**DOI:** 10.7705/biomedica.7138

**Published:** 2025-05-30

**Authors:** Dayan Crispín-Cruz, Alejandro Casas-Herrera, Camilo Rojas-Báez, Carlos Torres-Duque, Mauricio González-García

**Affiliations:** 1 Salud Respiratoria Comunitaria, Fundación Neumológica Colombiana, Bogotá, D. C., Colombia Fundación Neumológica Colombiana Fundación Neumológica Colombiana Bogotá, D. C. Colombia; 2 Dirección General, Fundación Neumológica Colombiana, Bogotá, D. C., Colombia Fundación Neumológica Colombiana Fundación Neumológica Colombiana Bogotá, D. C. Colombia; 3 Departamento de Investigación CINEUMO, Fundación Neumológica Colombiana, Bogotá, D. C., Colombia Fundación Neumológica Colombiana Fundación Neumológica Colombiana Bogotá, D. C. Colombia

**Keywords:** Asthma, diagnosis, child, disease prevention, quality of life, asma, diagnóstico, niño, prevención de enfermedades, calidad de vida

## Abstract

**Introduction.:**

International asthma management and prevention recommendations emphasize the importance of early and accurate diagnosis and adequate disease control. However, these aspects remain a serious concern, especially in children with low socioeconomic status.

**Objective.:**

To describe asthma prevalence, underdiagnosis, severity, and control among children with low socioeconomic status in Bogotá, Colombia.

**Materials and methods.:**

We conducted a cross-sectional study using the International Study of Asthma and Allergies Questionnaire in children aged 7-11 in two public schools. The children with affirmative answers in the questionnaire were evaluated clinically and functionally at the mobile health care unit. Asthma prevalence, underdiagnosis, control level, severity, and patients’ quality of life were assessed with validated instruments.

**Results.:**

We screened 920 schoolchildren with an age of 9.5 ± 1.1; 186 were evaluated clinically and functionally by spirometry, and 122 of them were diagnosed with asthma (17.2%). Underdiagnosis was 68%. Most patients had moderate and severe asthma, and 90% were cases of not well or very poorly controlled asthma.

**Conclusions.:**

Screening children in school settings is a suitable strategy for detecting asthma and reducing underdiagnosis in communities with low socioeconomic status and limited access to health care services. The asthma underdiagnosis and poor disease control were high.

## Resumen

**Introducción.:**

Las recomendaciones internacionales de manejo y prevención del asma enfatizan la importancia de un diagnóstico temprano y preciso y de un adecuado control de la enfermedad. Sin embargo, estos aspectos son motivo de preocupación, especialmente, en niños que viven en condiciones socioeconómicas desfavorables.

**Objetivo.:**

Estimar la prevalencia, el subdiagnóstico, la gravedad y el control del asma, en una población de niños de un entorno de bajo nivel socioeconómico en Bogotá, Colombia.

**Materiales y métodos.:**

Se realizó un estudio transversal por medio del *International Study of Asthma and Allergies Questionnaire* en niños de 7 a 11 años de dos escuelas públicas. Aquellos con alguna respuesta positiva en el cuestionario, fueron evaluados clínica y funcionalmente en la unidad móvil de atención médica.

**Resultados.:**

Se evaluaron 920 niños en edad escolar con una edad de 9,5 ± 1,1 años; 186 niños fueron evaluados clínica y funcionalmente por espirometría, y de ellos, 122 fueron diagnosticados con asma (17,2 %). El subdiagnóstico calculado fue del 68 %. El asma era moderada o grave en la mayoría de los casos y, en el 90 %, la enfermedad estaba parcialmente controlada o mal controlada.

**Conclusiones.:**

El tamizaje de niños en el entorno escolar es una estrategia adecuada para detectar el asma y reducir el subdiagnóstico en comunidades de bajo estrato socioeconómico y con acceso limitado a los servicios de atención en salud. Se obtuvieron porcentajes altos de subdiagnóstico y control deficiente del asma.

Asthma is a major focus in the political declaration of non-communicable diseases adopted by the United Nations General Assembly in 2018 ^1^. The international recommendations for asthma management and prevention emphasize the importance of early and accurate diagnosis and complete disease control, but these aspects remain a major concern, especially among children from low socioeconomic backgrounds ^2^^,^^3^.

Some studies suggest that low socioeconomic status is associated with higher severity and poorer control of the disease. This aspect directly impacts mortality, risk of exacerbations, in-hospital management, and, consequently, higher school absenteeism and even cessation of school activities ^4^^-^^6^. Numerous studies center on disease prevalence ^7^^,^^8^, and underdiagnosis has received less attention ^5^. Depending on the demographic characteristics and the definition of asthma diagnosis, underdiagnosis varies from 60 to 80% ^9^^,^^10^. The current prevalence of asthma in Colombia is 12% (95% CI: 10.513.7), and in 7- to 15-year-old children, it might be as high as 24%, with 43% of children (95% CI: 36.3-49.2) requiring emergency care or hospitalization in the past 12 months.

Among the approaches that might help to improve early asthma diagnosis, the case detection strategy identifies individuals with the disease who are symptomatic but undiagnosed. Most asthma-case detection strategies are implemented in schools with many advantages ^11^: the use of a single questionnaire will identify approximately one-quarter of the student body as possibly having asthma ^12^, and it is a unique opportunity to identify “high- risk” inner-city children with asthma who have many barriers to accessing healthcare services. Many programs combine screening with mobile-clinic care and pediatric asthma specialists ^13^^,^^14^.

To our knowledge, few studies have examined asthma underdiagnosis evidence based on a case detection strategy in low socioeconomic status populations considering school settings. Here, we report asthma prevalence, underdiagnosis, severity, and control in a low socioeconomic status population in Bogotá, Colombia.

## Materials and methods

We conducted a cross-sectional study for asthma screening in 7- to 11-year-old children in two public schools in Bogotá with a low socioeconomic status population, as defined by the classification of the *Departmento Administrativo Nacional de Estadística* (DANE). The schools were selected for convenience and easy accessibility.

Parents signed a consent form before answering the questionnaire and continuing with the clinical evaluation of their children, who also gave their written assent to participate in the study. The study was approved by the Ethics Committee at *Fundación Neumológica Colombiana*, regulated by the *Instituto Nacional de Vigilancia de Medicamentos y Alimentos* (Invima), the Colombian authority in charge of drugs and food surveillance and the national certification organ for ethics committees in Colombia.

### Procedures

We used the International Study of Asthma and Allergies Questionnaire (ISAAC) -validated in Spanish ^15^- for screening. We sent the questionnaire to be filled by parents at home, and a week later, they sent it back to the school. Then, the study group collected the forms and analyzed the information. The children with at least one affirmative answer in the questionnaire were candidates for a clinical evaluation.

The clinical evaluation and spirometry were performed on children at schools using a mobile healthcare unit specialized in respiratory diseases (called *Asmamóvil*). It included a small waiting area, a lung function testing area, and an examination room. Children were examined by an asthma medical doctor (a primary care physician trained by a pulmonologist), a respiratory nurse, and a physiotherapist.

### Asthma definitions

1. Self-reported lifetime asthma: affirmative answer to the question “Have your child ever had wheezing or whistling in the chest at any time in the past?”
2. Self-reported asthma in the last year: affirmative answer to the question “Have your child ever had wheezing or whistling in the chest in the past 12 months?”
3. Self-reported physician-diagnosed asthma: affirmative answer to the question “Has your child ever had asthma?”
4. Definitive asthma diagnosis: by the *Asmamóvil* physician based on clinical evaluation, symptoms, and spirometry, using the British Thorax Society Asthma Guidelines.
5. Asthma underdiagnosis was considered when participants had a definitive asthma diagnosis by the *Asmamóvil* physician and no previous asthma self-reported, diagnosed by a physician.

We used the National Asthma Education and Prevention (NAEEP) guidelines to classify severity as intermittent, mild, moderate, or severe persistent asthma and establish asthma control as well, not well, and very poorly controlled. The children’s control classification was established once they started treatment, considering that most had not been previously diagnosed with asthma. Although they might have consulted for respiratory symptoms before, they had no treatment plan.

Pulmonary function was assessed by baseline and post-bronchodilator spirometry using an EasyOne™ spirometer and following the American Thoracic Society-European Respiratory Society (ATS/ERS) guidelines ^16^. The body mass index (BMI) was applied according to the World Health Organization guidelines using BMI-for-age ^17^. The socioeconomic status was registered according to parents’ information and classified according to the *Departamento Administrativo Nacional de Estadística* (DANE), where level 1 is the lowest and level 6 is the highest. We considered levels 1, 2, and 3 as low socioeconomic status.

Quality of life was assessed using a validated Spanish version of the Pediatric Asthma Quality of Life Questionnaire (PAQLQ) ^18^. The three dimensions of the questionnaire -symptoms, activities, and emotions- were evaluated on a seven-point scale, with higher values indicating a better quality of life. Impairment categories are graded as follows: 1-3 = severe impairment, 4-6 = moderate impairment, and 7 = no impairment. Asthma severity and control, spirometry, and quality of life were outcomes assessed only in children with persistent asthma.

### Statistical analysis

We used mean and standard deviation or median and percentiles for continuous variables and proportions for categorical variables. Asthma prevalence and underdiagnosis were expressed as percentages. To calculate asthma prevalence, we used the number of children diagnosed by the *Asmamóvil* doctors and the number of those who returned the ISAAC questionnaire. The data was analyzed using SPSS™ statistics software, version 12.

## Results

We screened 920 schoolchildren between 7 and 11 years of age using the ISAAC questionnaire; 709 questionnaires were returned with a response rate of 77%. Among the respondents, 243 (34%) had at least one affirmative answer to the ISAAC questionnaire. Out of the 243, 140 (57%) reported wheezing in the chest at some time in the past (lifetime asthma), 51 (20%) reported wheezing in the chest in the past year, and 39 (16%) self-reported asthma diagnosed by a physician. All these cases were confirmed by a physician in the clinical evaluation.

**Figure 1:**
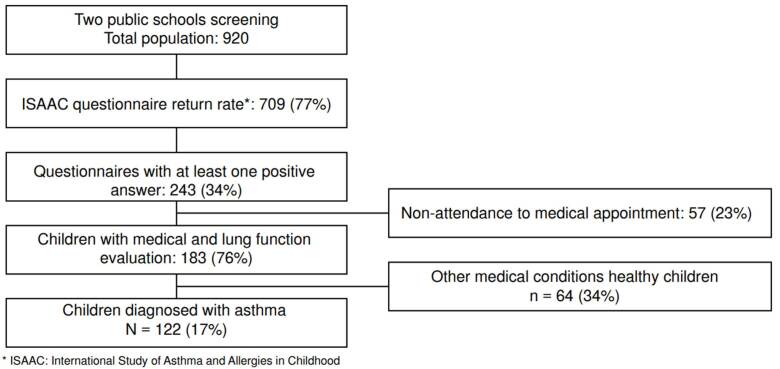
Study design flow chart from screening to final asthma diagnosis

From the 243 children with at least one affirmative answer, 186 (77%) underwent clinical evaluation and spirometry. Among these children, 122. were diagnosed with asthma by the *Asmamóvil* physician (17%). No differences in asthma prevalence were found between males and females. Eighty-three new asthma cases were diagnosed, showing a 68% underdiagnosis. Figure 1 describes the screening process throughout the final diagnosis.

Children with asthma had an age of 9.5 ± 1.1 years; most (80%) had low socioeconomic conditions, and (72%) a normal nutritional status. However, 23% were overweight (table 1). As for disease severity, 44% of the cases had moderate to severe asthma, with 90% with not well or very poorly controlled asthma. Only 27% of the children had used medication for asthma during the past year, and none used medication regularly. The mean value of pulmonary function was normal at baseline and a bronchodilator response was present in 14% of the sample. Quality of life was assessed in 113 children with persistent asthma. The PAQLQ results showed moderate impairment (median score = 4.3; interquartile range [IQR] = 3.8-5.8). Children with moderate quality of life impairment were 66.8% (n = 73), and 34% (n = 37) had severe impairment (table 1).

**Table 1:** Demographic data and clinical characteristics of children with asthma (N = 122)

Characteristic		Total n (%)
Age (years)		9.5 ± 1.1
Socioeconomic status		
	Low	98 (80)
	Very low	24 (20)
Gender		
	Male	67 (55)
	Female	55 (45)
Body mass index for age		
	Underweight	7 (5)
	Normal	88 (72)
	Overweight	27 (23)
Asthma severity		
	Intermittent	25 (20)
	Mild persistent	44 (36)
	Moderate persistent	48 (40)
	Severe persistent	5 (4)
Asthma control		
	Very poorly controlled	50 (45)
	Not well controlled	50 (45)
	Well controlled	13 (10)
	Treatment for asthma in the past year	33 (27)
Spirometry pre-bronchodilator Predicted		
	FEV_1_ (%)	95 ± 13
	FVC (%)	102 ± 14
	FEV_1_/FVC (%)	93 ± 6.7
Spirometry post-bronchodilator Predicted		
	FEV_1_ (%)	99 ± 15
	FVC (%)	103 ± 13
	FEV_1_/FVC (%)	95 ± 6.9
PAQLQ		4.3 (3.8-5.8)
	Moderate impairment	73 (66.8)
	Severe impairment	38 (34.2)

FEV_1_: forced expiratory volume in one second; FVC: forced vital capacity; PAQLQ: Pediatric Asthma Quality of Life Questionnaire

Medical evaluation confirmed other diagnoses in 186 children, such as, rhinitis (62%), atopic eczema (10%), adenoid hypertrophy (9%), and tonsil hypertrophy (7%), while 12% of the children were healthy.

## Discussion

Our main findings using the case detection strategy in school settings were asthma high prevalence, underdiagnosis, and high rates of asthma very poorly controlled in schoolchildren between 7 and 11 years of age from a low socioeconomic status in Bogotá.

The prevalence of asthma symptoms has been studied around the world with different results depending on the population. Using the ISAAC questionnaire, the mean prevalence for ever-asthma in Latin America is 13.4% in 6-7-year-old children and 12.6% in 13-14-year-old children ^19^. A study conducted in 6- to 18-year-old Peruvian students using a modified ISAAC questionnaire showed that 25.1% had been diagnosed with asthma by a health professional, and 16.7% reported wheezing in the past 12 months ^20^. In Colombia, Dennis *et al*. showed that 30.4% of 5- to 17-year-old children reported lifetime asthma, and 16.8% had asthma symptoms in the past year ^7^. We found that 57% reported wheezing in the chest at any time in life (lifetime asthma), and 20% reported wheezing in the chest during the past year (last year asthma), higher rates than in the above studies. Although we used the same questionnaire, we went further and performed a clinical and functional evaluation of these children. We found an asthma prevalence of 17.2%, similar to that reported in the literature.

Few population-based studies have reported asthma underdiagnosis in children. In Nigeria, a study with 45 children reported that only 24% were correctly diagnosed with asthma; the others were diagnosed with other diseases such as allergies, bronchitis (wheezing), pneumonia (chest infection), and tuberculosis ^21^.

In adult populations, the underdiagnosed rate in a study in The Netherlands was 66% ^22^, and in Colombia was 69.9%, which increased to 79% in 64-year-old or older subjects ^23^. In Jaipur, India, Gupta *et al*. suggested that asthma underdiagnosis and inadequate treatment resulted from patients’ treatment poor compliance and socioeconomic conditions, along with poor pediatricians’ knowledge, attitudes, and practices ^24^.

In our sample, children with asthma had normal spirometry values not related to symptom frequency or severity. This scenario was comparable to other studies in children with asthma whose pulmonary function parameters are frequently normal with or without positive bronchodilator change. In 630 children who completed spirometry, Murray *et al*. showed that the FEV_1_/FVC was less than 70% only in ten (2%), two of whom had current asthma diagnoses. The bronchodilator response was positive in 54 (9%) out of 624 children ^25^.

As for asthma severity, we found that most of the participants have moderate or severe persistent asthma with extremely high rates of no control of the disease, meaning significant daily symptoms, risk of exacerbation, and poor disease-related quality of life, which is consistent with other studies ^2^. As expected, a high proportion of children had atopic dermatitis or allergic rhinitis. This information should be considered to establish adequate treatment for these conditions and improve asthma control ^26^.

All patients experienced some degree of quality-of-life impairment, mostly moderate, as indicated by the PAQLQ scores. These results are similar to those of Dueñas *et al*., who evaluated baseline quality of life in children with asthma and reported a median score of 4.6 ± 1.3 in the PAQLQ ^27^.

The limited healthcare services access, the inadequate understanding of international guidelines for asthma diagnosis by primary care physicians, or a combination of these factors might explain asthma underdiagnosis in our children ^22^. Understanding prevalence and underdiagnosed rates may help to develop a comprehensive program for chronic respiratory disease diagnosis, management, and follow-up in children. As for sustainability, school-based asthma screening is not cost-effective, and the approach should lean towards screening for previously diagnosed patients with poorly controlled asthma ^28^. However, more studies are needed to analyze the cost-benefit in our population.

We have the challenge to provide better training for healthcare professionals based on national and international asthma diagnosis and treatment guidelines. Whereas the school setting might be a good option for asthma-timely screening and diagnosis, families and the private and public health sectors’ cooperation and involvement are also crucial to guarantee health equity and proper follow-up of chronic diseases such as asthma.

A case-detection strategy with clinical confirmation to identify asthma prevalence and underdiagnosis in children belonging to low-income populations is the main strength of our study. We considered that the 77% questionnaire response rate was comparable with the over 60% response rates reported in the literature. These results reflect our effective communication with the school head, the staff, and the teachers involved in the study’s in-school activities. To correlate our findings with other studies, we used an international questionnaire and validated clinical management guidelines for asthma research. Finally, our results broaden the existing literature on asthma prevalence and underdiagnosis, given the scarce studies in Latin America specifically focused on children of low socioeconomic status.

As for study limitations, we should mention the risk of selection bias: the schools were chosen due to their convenient location and did not necessarily represent the general population. Also, survey information always faces the risk of bias since respondents may not know or recognize asthma-related symptoms. There was a high probability that in the group of non-respondents, the majority of the children were asymptomatic, and their parents were not motivated to respond to the questionnaire, which may have resulted in asthma prevalence overestimation in the study.

We applied a case-detection strategy in children of low socioeconomic status in Bogotá, and we identified a high prevalence of asthma and underdiagnosis with high rates of asthma poorly controlled. Screening children in school settings is a suitable strategy for detecting asthma and reducing underdiagnosis, inequity, and limited access to healthcare services in low-socioeconomic status communities.
